# Association of Maternal Hypothyroidism With Cardiovascular Diseases in the Offspring

**DOI:** 10.3389/fendo.2021.739629

**Published:** 2021-08-31

**Authors:** Maohua Miao, Hui Liu, Wei Yuan, Nicolas Madsen, Yongfu Yu, Krisztina D. László, Hong Liang, Honglei Ji, Jiong Li

**Affiliations:** ^1^National Health Commission Key Laboratory of Reproduction Regulation (Shanghai Institute for Biomedical and Pharmaceutical Technologies), Fudan University, Shanghai, China; ^2^Department of Clinical Epidemiology, Aarhus University, Aarhus, Denmark; ^3^Medical Informatics Center, Peking University, Beijing, China; ^4^Acute Care Cardiology Unit, Department of Pediatrics, University of Cincinnati, Cincinnati, OH, United States; ^5^Department of Public Health Sciences, Karolinska Institutet, Stockholm, Sweden

**Keywords:** hypothyroidism, *in utero* exposure, nationwide cohort study, cardiovascular disease, register-based

## Abstract

**Background:**

No previous study has examined the effect of maternal hypothyroidism on a broad spectrum of cardiovascular disease (CVD) endpoints in the offspring.

**Methods:**

A nationwide population-based cohort study based on the linkage of several Danish nationwide registries was conducted to explore whether maternal hypothyroidism is associated with offspring’s CVD. Altogether 1,041,448 singletons born between the 1st of January 1978 and the 31st of December 1998 were investigated from the age of 8 years to the 31st of December 2016. Exposure was maternal diagnosis of hypothyroidism across lifespan and the outcome of interest was a CVD diagnosis in the offspring. Cox regression models were performed to estimate the hazard ratios (HRs) of CVD.

**Results:**

Offspring born to mothers with hypothyroidism had an increased risk of CVD (hazard ratios (HR)=1.23, 95% confidence interval (CI): 1.12-1.35), and of several subcategories of CVD including hypertension, arrhythmia, and acute myocardial infarction in offspring. The magnitude of association was the most pronounced in an exposure occur during pregnancy (HR=1.71, 95% CI: 1.10-2.67), which is consistent across all the subgroup analysis, including sibling analysis.

**Conclusions:**

Maternal hypothyroidism is associated with an increased risk of CVD in offspring. Thyroid hormone insufficiency during pregnancy may predominantly contribute to the observed associations; however, the effects of a shared genetic background and a time-stable familial environment/lifestyle factors cannot be excluded.

## Introduction

Cardiovascular disease (CVD) is the leading cause of death and disability worldwide (1, 2). Although a number of environmental and genetic risk factors have been identified, the aetiology of a large proportion of CVD remains to be elucidated, in particular those occurring at a younger age (1, 3). An increasing body of evidence has supported the hypothesis of “foetal origins” of CVD, suggesting that adverse conditions *in utero* may play an important role in the development of CVD later in life (4).

Hypothyroidism, an endocrine disorder characterized by thyroid deficiency, is a common condition that can complicate pregnancy, with a prevalence of 2%-5% (5). Thyroid hormones are involved in multiple physical processes during intrauterine life; however, the foetus does not produce thyroid hormones until mid-gestation. Therefore, sufficient expression of maternal thyroid hormones is crucial for foetal development (6). Overt hypothyroidism is associated with multiple adverse pregnancy outcomes, such as preterm birth, low birth weight, and neurodevelopmental disorders (7–10). Maternal hypothyroidism may also affect the development of the foetal cardiovascular system (11, 12). Adequate maternal thyroid function is necessary for the proper expression of both structural and regulatory genes in cardiomyocytes of foetal rats (11). Human study has also indicated an association between maternal hypothyroidism and congenital heart disease (12). Furthermore, study has also suggested a programming effect of maternal thyroid function on blood pressure of offspring at the age of 20 years (13). However, to our knowledge, no previous study has examined the effect of maternal hypothyroidism on a broad spectrum of CVD endpoints at elder ages.

We conducted a nationwide population-based cohort study to examine the association between maternal hypothyroidism and the risk of CVD in offspring using Danish registry data. In the present study, we focused on CVD in adolescents and adults, since the association with congenital heart disease has been reported (12). The large sample size and the opportunity for a long-term follow-up allowed us to examine effect of maternal hypothyroidism occurring during pregnancy, as well as in other time windows.

## Material and Methods

### Study Population

**Figure 1:**
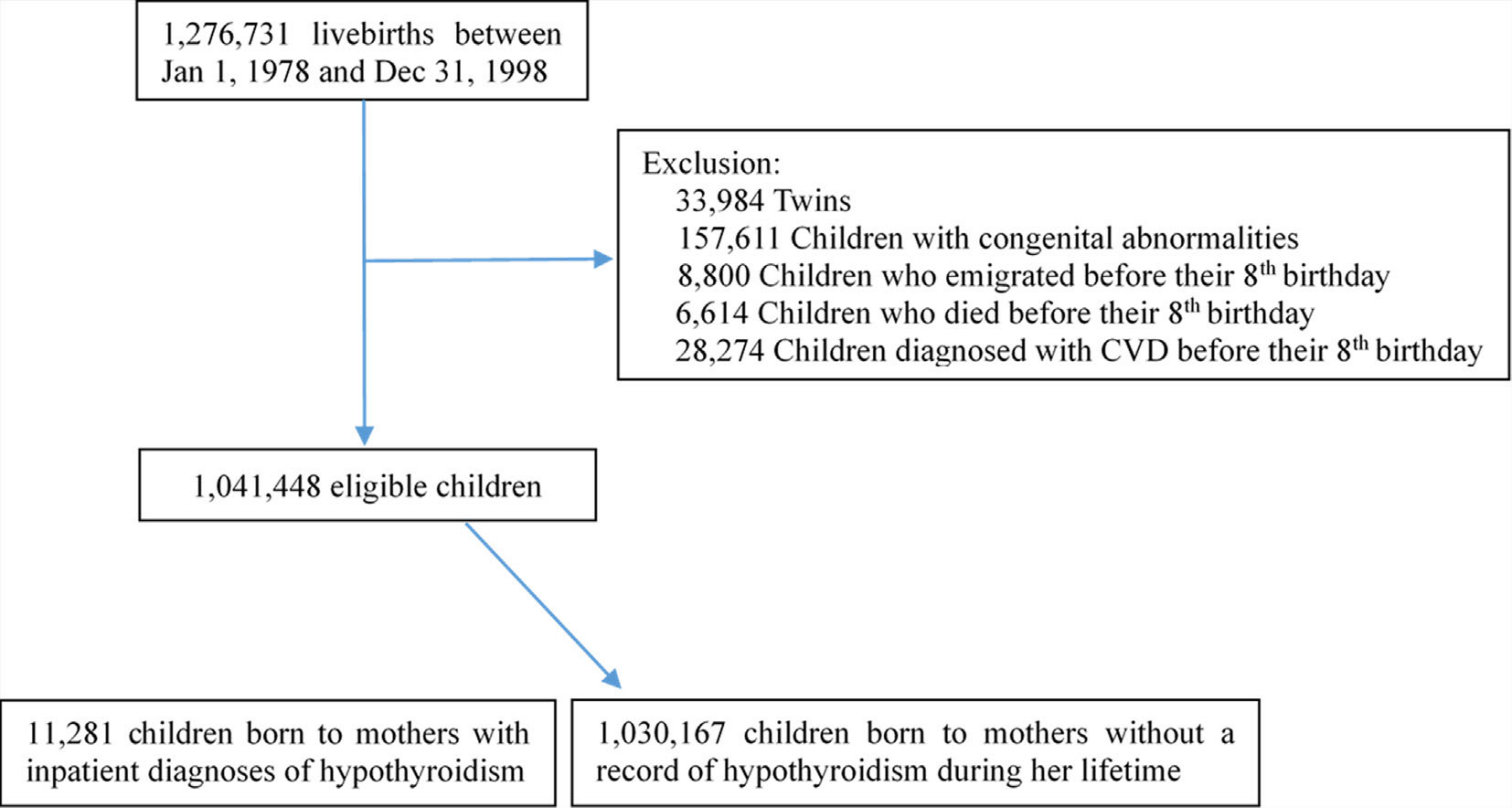
Flow chart illustrating the identification of the study population.

We constructed a population-based cohort by linking several Danish population-based registries through the unique personal identification number assigned to all residents. We included all live singleton births born in Denmark from the 1^st^ of January 1978 to the 31^st^ of December 1998 identified from the Danish Medical Birth Register (DMBR) (14). Follow-up commenced at the age of 8 years and offspring born after 1998 were excluded to make sure that study subjects of offspring could be followed for at least 10 years at the end of follow-up (31^st^ of December, 2016). We also excluded offspring with congenital anomalies that may have a higher risk of CVD due to congenital heart disease or medical intervention to treat for other anomalies (15). Among the 1,276,731 live births during the study period, we included 1,041,448 singleton births in the final study population after excluding twins (33,984), offspring with congenital anomalies (157,611), offspring emigrated (8,800) or died (6,614) before their 8^th^ birthday, and offspring who had a CVD diagnosis before their 8^th^ birthday (28,274) (**Figure 1**). All offspring were followed from the age of 8 until onset of CVD, death, emigration, or December 31, 2016, whichever came first. The longest follow-up was 38 years. The study was approved by the Danish Data Protection Agency (registration no. 2013-41-2569).

### Ascertainment of Maternal Hypothyroidism

We classified offspring as exposed, if their mothers had at least one inpatient diagnosis of hypothyroidism (ICD-8 codes 243-244, and ICD-10 codes E03 and E89.0) between 1977 and 2016 in the Danish National Patient Registry (DNPR) (16), which contained information on all hospital admissions in the country since 1977. Outpatient diagnosis of maternal hypothyroidism was only included in a subgroup of offspring born after 1996 since the register did not contain outpatient care information until 1995. Exposed offspring were classified according to the date of the first diagnosis of maternal hypothyroidism: before conception, during pregnancy, up to five years after delivery, and more than five years after delivery. The start date of pregnancy was estimated by subtracting the gestational age from the date of birth. Offspring from mothers that had no hospital record of hypothyroidism in the DNPR were allocated to the unexposed group. Paternal hypothyroidism was identified with the same strategy in the DNPR.

### Ascertainment of CVD

Cases of CVD were identified in the inpatient and outpatient components of the DNPR, as well as in the Cause of Death Registry, defined by International Classification of Diseases (ICD) codes 390-459 (ICD-8) and I00-I78 (ICD-10). The dates of the earliest diagnosis of any type of CVD were identified. Furthermore, the most prevalent CVD subtypes were also identified, i.e. hypertension, ischemic heart disease, arrhythmia (including supraventricular arrhythmias and ventricular arrhythmias), atrial fibrillation, stroke, and acute myocardial infarction (for ICD codes see **Supplementary Table 1**). For the sub-analysis with hypertension, we also considered prescriptions of hypertensive medications from the Danish National Prescription Registry (17) with the ATC codes C02, C03, C07, C08, and C09; those with either a hospital record of hypertension or with at least two anti-hypertensive prescriptions were considered to have hypertension. The time of the first diagnosis or first dispensed prescription, whichever came first, was used to define the onset of hypertension.

### Ascertainment of Covariates

We obtained information on maternal age at childbirth, parity, the offspring’s date of birth, gender, gestational age, birth weight, and Apgar score at five minutes from the DMBR (18). Information on maternal cohabitation, maternal employment and year of maternal education were obtained from the Danish Civil Registration System (19) and the Integrated Database for Longitudinal Labor Market Research (20). Maternal comorbidities that have been reported to be associated with both the exposure and outcome of interest, i.e. maternal CVD and diabetes (ICD8: 249-250, ICD10: E10-E14), were also retrieved from the DNPR.

We replaced missing and invalid values (<154 or >315 days) of gestational age with the median of all the singleton live births. Missing values on parity were imputed based on the number of older siblings in the DMBR. In case of a missing value for maternal cohabitation and education at conception, we used information on these variables from the previous or the subsequent three years, if available.

### Statistical Analysis

We first described the demographic characteristics of the study population and calculated the age-specific CVD incidence rates for the exposed and the unexposed cohorts. We used Cox proportional hazard models to examine the association between maternal hypothyroidism and CVD, as well as the six most prevalent subcategories of CVD, including hypertension, ischemic heart disease, arrhythmia, atrial fibrillation, stroke, and acute myocardial infarction. We further evaluated the risks according to the time of the first diagnosis of maternal hypothyroidism, i.e. before conception, during pregnancy, up to five years after delivery, and more than five years after delivery. Similar analyses were performed for hypertension, but not for the other CVD subcategories, given the limited cases. Covariates including year of birth, gender, parity, maternal age at childbirth, education, and employment were adjusted for in the cox regression model.

To examine the role of shared genetic and time-stable familial environment/lifestyle factors, we performed parallel analyses with paternal hypothyroidism, as well as a sibling analysis. The latter was conducted among those with more than one sibling using stratified Cox model cluster by identity of mother.

We conducted several sensitivity analyses to test the robustness of our findings. First, we restricted the analyses to children born between 1996 and 1998, who had an outpatient diagnosis of maternal hypothyroidism to reveal any changes in the estimates of the association. Second, to evaluate the effect of co-existing diseases, we described the distribution of the comorbid diseases at the first diagnosis of hypothyroidism and added the main co-existing diseases as covariates in the Cox regression model where appropriate. Third, we stratified the analyses by maternal CVD and diabetes to see whether they play a major role in the observed association. Fourth, to examine whether the effect could be modified by maternal age at delivery and education, we conducted stratified analyses according to these variables. Stratified analyses on offspring’s gender and age of CVD diagnosis were also performed to examine whether the associations were gender or age-specific. Fifth, to examine the potential mediating effect of adverse birth outcome, we added preterm birth, birth weight, and Apgar score as covariates to identify any changes in the estimates of the association. Finally, we repeated the above analyses 1) using data without any replacement for missing values; 2) among those with Danish origin; 3) excluding mothers who were diagnosed with hyperthyroidism before hypothyroidism, to test the stability of the results.

All analyses were conducted using the Survival package in R.

## Results

There were 11,281 study participants born to mothers with hypothyroidism (1.1%). **Table 1** shows the characteristics of mothers and offspring according to exposure status. Compared to the unexposed offspring, exposed offspring were more likely to have higher birthweight, be born preterm, born to mothers who were older, had a higher education, and had CVD (20.5% *vs*. 45.9%) or diabetes (2.6 *vs*. 11.9%).

**Table 1:** Baseline characteristics of the study population according to maternal hypothyroidism, n (%).

Characteristic	Maternal hypothyroidism	No maternal hypothyroidism
	(N=11,281)	(N=1030,167)
Year of birth		
1978-1982	3214 (28.5)	229,796 (22.3)
1983-1987	2650 (23.5)	216,111 (21.0)
1988-1992	2566 (22.7)	256,809 (24.9)
1993-1998	2851 (25.3)	327,451 (31.8)
Sex		
Male	5746 (51.0)	521,034 (50.6)
Female	5520 (49.0)	508,178 (49.4)
Birth weight (g)		
<2500	455 (4.0)	36,424 (3.5)
2500-3250	3532 (31.3)	313,479 (30.4)
3251-3999	5381 (47.7)	508,545 (49.4)
≥4000	1862 (16.5)	166,668 (16.2)
Unknown	51 (0.5)	5,051 (0.5)
Preterm birth^a^		
Yes	503 (4.5)	38,420 (3.7)
No	10,778 (95.5)	991,747 (96.3)
Apgar score at 5 minutes		
0-7	118 (1.0)	12,926 (1.3)
8-9	558 (4.9)	49,362 (4.8)
10	10,482 (93.0)	956,773 (92.8)
Unknown	123 (1.1)	11,106 (1.1)
Parity		
1	5133 (45.6)	497,033 (48.2)
2	3972 (35.2)	367,252 (35.7)
≥3	2171 (19.2)	165,582 (16.1)
Unknown	5 (0.0)	300 (0.0)
Maternal age at childbirth (years)		
12-19	412 (3.7)	33,214 (3.2)
20-24	2777 (24.6)	242,017 (23.5)
25-29	4098 (36.3)	407,976 (39.6)
30-34	2774 (24.6)	255,681 (24.8)
≥35	1220 (10.8)	91,279 (8.9)
Maternal education (years)		
≤9	1537 (13.6)	176,750 (17.2)
10-14	3602 (31.9)	396,890 (38.5)
≥15	3934 (34.9)	302,811 (29.4)
Unknown	147 (1.3)	9088 (0.9)
Maternal smoking in the first trimester (since 1991)		
Yes	1292 (33.4)	131,505 (30.3)
No	2388 (61.7)	283,825 (65.3)
Unknown	191 (4.9)	19,352 (4.5)
Maternal employment		
Not in the labour market	3774 (33.5)	286,313 (27.8)
Blue collar	1910 (16.9)	178,054 (17.3)
White collar	2837 (25.1)	313,690 (30.5)
Executive	1350 (12.0)	153,724 (14.9)
Unknown	28 (0.2)	28 (0.0)
Maternal cohabitation at childbirth		
Single	4688 (41.6)	443,751 (43.1)
Cohabitating	6579 (58.3)	585,293 (56.8)
Unknown	0 (0.0)	56 (0.0)
Maternal diabetes		
Yes	1347 (11.9)	27,259 (2.6)
No	9934 (88.1)	1002,908 (97.4)
Maternal CVD^a^		
Yes	5181 (45.9)	211,133 (20.5)
No	6100 (54.1)	819,034 (79.5)
Paternal age at childbirth (years)^b^		
13-24	1494 (13.2)	121,600 (11.8)
25-29	3624 (32.1)	347,149 (33.7)
30-34	3459 (30.7)	330,680 (32.1)
≥35	2635 (23.4)	224,816 (21.8)

CVD, cardiovascular diseases.

a Defined as a gestational age ≤258 days.

b There are 5944 (0.57%) fathers with missing CPR number.

During the follow-up, which was starting from 8 years, 102,394 offspring (9.8%) emigrated, 5,735 died (0.6%), and 30,860 were diagnosed with CVD, yielding a CVD incidence rate of 25.9 per 1000 person-years. The median follow-up time was 18.2 years (interquartile range: 13.8 to 24.0 years), and the median age at the first diagnosis of CVD was 23.2 years (interquartile range: 18.8 to 28.5 years).

We observed consistently higher incidence rates of CVD in the exposed group than the unexposed, despite the offspring’s ages (**Supplement Figure S1**). Compared with the unexposed offspring, the HR of CVD for the exposed was 1.23 (95% confidence interval (CI), 1.12-1.35) after adjusting for year of birth, sex, parity, maternal age at childbirth, education, and employment. When we restricted the analyses to children born between 1996 and 1998 who had outpatient diagnosis of maternal hypothyroidism, the association remained (HR=1.41, 95% CI, 0.90-2.21), although the estimate was less accurate due to the largely decreased sample size. The risks were similar for several main CVD subcategories including hypertension, arrhythmia, and acute myocardial infarction, as well as a weaker association with ischaemic heart disease (**Table 2**).

**Table 2:** Hazard ratios and 95% confidence intervals for offspring’s CVD according to exposure of maternal and paternal hypothyroidism.

	No. of events	Incidence rate (1/1000 person-years)	HR (95% CI)	
			Crude	Adjusted^b^
**Maternal hypothyroidism** ^a^				
CVD				
Unexposed	30411	24.29	1.00 (reference)	1.00 (reference)
Exposed	449	32.73	1.29 (1.18,1.42)	1.23 (1.12,1.35)
Hypertension (identified through. patient registry)				
Unexposed	5937	5.41	1.00 (reference)	1.00 (reference)
Exposed	105	8.57	1.51 (1.25,1.84)	1.39 (1.14,1.69)
Hypertension (identified through. patient registry and prescription registry)				
Unexposed	36363	29.33	1.00 (reference)	1.00 (reference)
Exposed	661	48.30	1.61 (1.49,1.73)	1.47 (1.36,1.59)
Ischaemic heart disease				
Unexposed	2413	2.20	1.00 (reference)	1.00 (reference)
Exposed	42	3.45	1.49 (1.10,2.03)	1.34 (0.98,1.82)
Arrhythmia				
Unexposed	10817	8.14	1.00 (reference)	1.00 (reference)
Exposed	155	10.78	1.27 (1.08,1.48)	1.22 (1.04,1.44)
Atrial fibrillation				
Unexposed	1603	1.36	1.00 (reference)	1.00 (reference)
Exposed	23	1.82	1.25 (0.83,1.88)	1.20 (0.79,1.81)
Stroke				
Unexposed	3169	2.61	1.00 (reference)	1.00 (reference)
Exposed	41	2.74	1.13 (0.83,1.53)	1.04 (0.76,1.43)
Acute myocardial infarction				
Unexposed	489	0.49	1.00 (reference)	1.00 (reference)
Exposed	13	1.18	2.25 (1.30,3.90)	2.02 (1.16,3.50)
**Paternal hypothyroidism** ^a^				
CVD				
Unexposed	30768	24.37	1.00 (reference)	1.00 (reference)
Exposed	92	31.94	1.28 (1.05,1.58)	1.21 (0.99,1.49)
Hypertension				
Unexposed	36904	29.51	1.00 (reference)	1.00 (reference)
Exposed	120	41.11	1.42 (1.18,1.70)	1.25 (1.04,1.49)

CVD, cardiovascular disease; HR, hazard ratio; CI, confidence intervals.

a maternal(paternal) diagnosis of hypothyroidism across lifespan.

b adjusted for year of birth, sex, parity, maternal age at childbirth, maternal education, and maternal employment.

We observed a more pronounced association when the exposure occurred during pregnancy (HR=1.71, 95% CI, 1.10-2.67). No association was observed for exposure occurring before pregnancy, or up to five years after pregnancy, probably due to lack of statistical power. Notably, an association was also observed for maternal hypothyroidism diagnosed more than five years after delivery (HR=1.22, 95% CI, 1.11-1.35). Similar patterns were observed for hypertension, the most common subcategory of CVD in our study population (**Table 3**).

**Table 3:** Hazard ratios and 95% confidence intervals for offspring’s CVD according to maternal hypothyroidism in different exposure windows.

	No. of events	Incidence rate (1/1000 person-years)	HR (95% CI)	
			Crude	Adjusted^b^
**CVD**				
Unexposed	30411	24.29	1.00 (reference)	1.00 (reference)
Exposed^a^	449	32.73	1.29 (1.18,1.42)	1.23 (1.12,1.35)
During Pregnancy	19	38.95	1.60 (1.02,2.50)	1.71 (1.10,2.67)
Only Before Conception	12	18.66	1.09 (0.62,1.92)	1.23 (0.69,2.18)
Only During First 5 Years after Delivery	11	18.77	0.92 (0.51,1.65)	0.90 (0.49,1.66)
Only More Than 5 Years after Delivery	407	33.56	1.30 (1.18,1.43)	1.22 (1.11,1.35)
Only More Than 5 Years after Delivery (with additional adjustment for co-existing disease^c^)				1.19 (1.04,1.37)
**Hypertension** (identified through patient registry and prescription registry)				
Unexposed	36363	29.33	1.00 (reference)	1.00 (reference)
Exposed^a^	661	48.30	1.61 (1.49,1.73)	1.47 (1.36,1.59)
During Pregnancy	24	44.22	1.67 (1.12,2.50)	1.81 (1.21,2.69)
Only Before Concept	15	30.12	1.15 (0.69,1.90)	1.36 (0.81,2.26)
Only During 0-5 Years after Delivery	17	30.08	1.17 (0.73,1.88)	1.39 (0.87,2.24)
Only More Than 5 Years after Delivery^c^	605	49.86	1.63 (1.51,1.77)	1.47 (1.35,1.59)

CVD, cardiovascular disease; HR, hazard ratio; CI, confidence intervals.

a maternal diagnosis of hypothyroidism across lifespan.

b adjusted for year of birth, sex, maternal parity, maternal age at childbirth, maternal education, and maternal employment.

c Includes neoplasms, circulatory, digestive, and genitourinary diseases.

Parallel analyses with paternal hypothyroidism exposure also suggested an increased risk for CVD (HR=1.21, 95% CI, 0.99-1.49 for overall CV, and HR=1.25, 95% CI, 1.04-1.49 for hypertension, **Table 2**).

In the comparison between siblings with and without exposure of maternal hypothyroidism during pregnancy, we included 313,905 families, corresponding to 709.474 offspring. We observed similar associations as the main analysis (HR=1.73, 95% CI, 0.96-3.10 for overall CVD, and HR=2.01, 95% CI, 1.20-3.36 for hypertension) (**Table 4**).

**Table 4:** Hazard ratios and 95% confidence intervals for offspring’s CVD according to maternal hypothyroidism during pregnancy in a sibling-matched analysis.

	No. of events	Incidence rate (1/1000 person-years)	HR (95% CI)	
			Crude	Adjusted^b^
**CVDs**				
Unexposed	19786	25.69	1.00 (reference)	1.00 (reference)
Exposed^a^	13	41.28	1.75 (1.02,3.04)	1.73 (0.96,3.10)
**Hypertension** (identified through patient registry and prescription registry)				
Unexposed	23089	32.46	1.00 (reference)	1.00 (reference)
Exposed^a^	15	53.16	1.79 (1.08,2.97)	2.01 (1.20,3.36)

CVD, cardiovascular disease; HR, hazard ratio; CI, confidence intervals.

a maternal diagnosis of hypothyroidism during pregnancy.

b adjusted for: year of birth, sex, parity, maternal age at childbirth.

For the diagnosis of maternal hypothyroidism during pregnancy, most of them (69%, **Supplementary Table 2**) were given as a co-existing disease at childbirth-related medical process, which indicate an equal chance for all pregnant women, and are expected to produce less detection bias. For maternal hypothyroidism occurring after delivery, a large percentage was identified with a co-existing diagnosis of the digestive system (10.88%), the genitourinary system (10.74%) or neoplasm (7.22%) (**Supplementary Table 2**). We added these co-existing diseases as covariates in the analysis of exposure after delivery, the association decreased slightly but remain statistically significant (**Table 3**).

**Figure 2:**
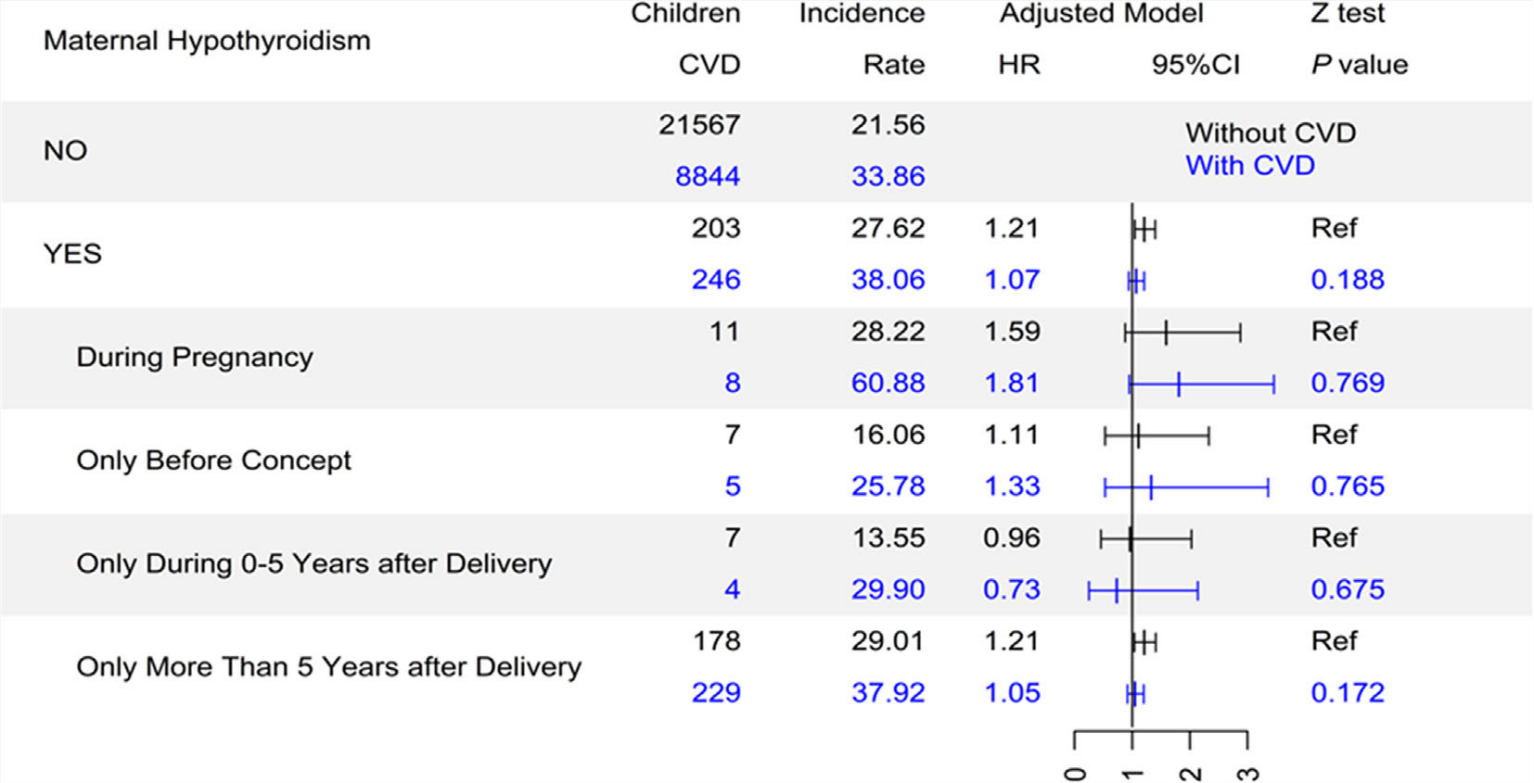
The association between maternal hypothyroidism and offspring’s CVD stratified by maternal CVD. CVD, cardiovascular disease; Incidence rate, incidence rate per 1000 person-years; HR, hazard ratio; CI, confidence interval; Ref, reference group.

We observed decreased associations between maternal hypothyroidism exposure and CVD in the strata of offspring with maternal CVD (**Figure 2**) and Diabetes (**Supplementary Figure S2**), but no such decreases were observed for the exposure during pregnancy. Stratification analyses by maternal age at delivery, maternal education, the offspring’s gender, and age at CVD diagnosis did not show evidence of effect modification by these factors, except that offspring who had CVD diagnosed before the age of 17 seemed more likely to be born to mothers with hypothyroid diagnosed before conception (**Supplementary Figures S3–S6**).

Adding preterm birth, birth weight, and Apgar score at five minutes as covariates did not change the associations substantially, even for those exposed during pregnancy. Using data without any missing value replacement, restricting the analysis to mothers of Danish origin, and excluding mothers (68) who were diagnosed with hyperthyroidism before hypothyroidism, yielded similar results as the main analyses (data not shown).

## Discussion

In this population-based cohort study, we observed an association between maternal hypothyroidism and an increased risk of CVD in offspring from adolescence to mid-adulthood. The most consistent association was observed for offspring exposed to maternal hypothyroidism during pregnancy, which was also supported by sibling analysis. Notably, a weaker association was also observed among offspring born to fathers who had a diagnosis of hypothyroidism across their lifespan, as well as to mothers who developed hypothyroidism more than five years after delivery.

Earlier studies have documented an association between maternal hypothyroidism and an increased risk of congenital heart diseases (13). Two prospective studies have also examined associations between maternal hypothyroidism and hypertension (13, 21). In the study by Rytter et al., offspring of subclinical hypothyroid mothers had higher systolic blood pressure and a tendency towards higher diastolic blood pressure at the age of 20 (13). Our results added to the evidence that maternal hypothyroidism is associated with a wider spectrum of CVD other than hypertension in the offspring.

The consistent findings on the increased risk of CVD following *in utero* exposure to maternal hypothyroidism in our study suggested that thyroid hormone deficiency during *in utero* development might predispose offspring to an adverse cardiovascular development. Thyroid hormones are involved in multiple physical processes, particularly during the intrauterine life. The foetus wholly relies on maternal thyroxin until the onset of foetal thyroid hormone secretion at 14-18 gestational weeks, which coincides with important developmental events, including cardiovascular development (6). Experimental studies in mice have shown that thyroid hormones are involved in the development of specific hypothalamic centres governing cardiovascular function (22). Animal models of maternal hypothyroidism have also revealed a variety of anomalies, which are involved in cardiac function, including the renin-angiotensin system, beta-adrenergic system, and endothelial functions (23, 24). Additionally, epigenetic mechanisms are also plausible since maternal hypothyroidism has been observed to cause histone modifications and alteration in DNA methylation (25, 26).

Hypothyroidism is associated with later occurrence of CVD in human adults (27, 28). which made us hypothesize that the two diseases may share similar personal genetic backgrounds. This hypothesis provides an explanation to our findings including: 1) weak associations were also observed among those with only paternal exposure, as well as among those with maternal hypothyroidism exposure more than 5 years after delivery; and 2) a decreased risk was observed in the stratum with maternal CVD. However, it cannot be excluded that shared familial environmental factors for hypothyroidism and CVD may also play a role.

Maternal hypothyroidism can result in adverse birth outcomes in offspring (29). Hence, it is plausible that the association between maternal hypothyroidism and risk of CVD in offspring is mediated in part through increased risks of adverse birth outcomes, which are independently associated with increased risk for CVD in later life. However, this hypothesis was not supported since the estimates corresponding to the association between maternal hypothyroidism and CVD did not change substantially, when we added preterm birth, birth weight, and Apgar score as covariates in our multivariate model.

The present study has a number of strengths. The large population-based study with virtually complete follow-up makes bias due to non-response or loss to follow-up unlikely. Due to the large sample size, we were able to examine less frequent outcomes such as CVD in adolescents and young adults. Additionally, we were able to evaluate the possible genetic effects through the inclusion of paternal hypothyroidism as a negative control and stratification by maternal CVD comorbidities.

We acknowledge several important limitations. First, we identified exposed subjects through inpatient record of hypothyroidism in the main analyses. Since the symptoms of hypothyroidism can be mild and unspecific, our study might be overrepresented by more severe hypothyroid cases and cases with other co-existing diseases (30). We evaluated the potential bias through a sub-analysis among those with outpatient data of maternal hypothyroidism and additional adjusting the co-existing disease, and found similar associations, suggesting a minimal effect of detection bias. In addition, the most consistent association was observed among those with maternal exposure during pregnancy, for whom the bias would be less since all pregnant women would be recruited as an inpatient for delivery. Despite these, a more accurate method of exposure identification is needed to verify our findings. Second, the maximum age of follow-up in our study was 38 years, and association for offspring of older ages are not known. However, the consistently higher incidence rates of the exposed group observed across all ages indicated that the risk might remain later in life. Third, offspring who had congenital anomalies were excluded in our study. This may have introduced a bias toward null, since hypothyroidism in pregnant mothers may lead to congenital anomalies including those with a high risk on CVD. Fourth, we do not have enough data on medication to examine the potential protective effect of thyroid hormone treatment since the prescription registry was not established in Denmark until 1995. Similarly, the information on how well-controlled maternal hypothyroidism, especially during pregnancy, was not available in the present study. These mothers would be classified as unexposed; however the estimates would probably be biased toward null. Finally, some important covariates, such as BMI of offspring, were not routinely collected in Danish medical registry, and thus their potential confounding effect cannot be evaluated in the present study.

In conclusion, maternal hypothyroidism may have an adverse effect on offspring’s cardiovascular health from adolescence to mid-adulthood, particularly when the exposure occurs during pregnancy. The effects of genetic susceptibility or common familial environmental factors cannot be fully excluded, due to the associations observed for paternal hypothyroidism as well as for maternal exposure more than 5 years after delivery. Although the magnitude of the association between hypothyroidism and an increased CVD risk is small, it may translate into a substantial increase in risk at the population level given the high prevalence of hypothyroidism.

## Data Availability Statement

All data used in this article were obtained from the Danish national registers, including Danish Medical Birth Register, Danish National Patient Register, and others. Due to restrictions related to Danish law and protecting patient privacy, the data used in this study can only be made available through a trusted third party, Statistics Denmark (https://www.dst.dk/en/kontakt). University-based Danish scientific organisations can be authorized to work with data within Statistics Denmark and such organisation can provide access to individual scientists inside and outside of Denmark. Researchers can apply for access to these data when the request is approved by the Danish Data Protection Agency: https://www.datatilsynet.dk.

## Ethics Statement

The studies involving human participants were reviewed and approved by the Danish Data Protection Agency. Written informed consent from the participants’ legal guardian/next of kin was not required to participate in this study in accordance with the national legislation and the institutional requirements.

## Author Contributions

MM: Conceptualization, funding acquisition, writing-original draft, and writing-review and editing. HuL: Formal analysis, funding acquisition, and writing-review and editing. WY: Writing-review and editing. NM: Writing-review and editing. YY: Methodology and writing-review and editing. KL: Writing-review and editing. HoL: Writing-review and editing. HJ: Writing-review and editing. JL: Conceptualization, funding acquisition, and writing-review and editing. All authors contributed to the interpretation of the results and critical revision of the manuscript, and gave final approval and agree to be accountable for all aspects of work ensuring integrity and accuracy. All authors contributed to the article and approved the submitted version.

## Funding

This work was supported by grants from the National Key Research and Development Program (2016YFC1000505, 2018YFC1002801); Independent Research Fund Denmark (DFF-6110-00019B, DFF-9039-000010B); the Lundbeck Foundation (R232-2016-2462 and R265-2017-4069); the Nordic Cancer Union(R275-A15770); the Karen Elise Jensens Fond (2016); Novo Nordisk Fonden (NNF18OC0052029), Innovation-oriented Science and Technology Grant from NHC Key Laboratory of Reproduction Regulation (CX2017-06), the National Nature Science Foundation of China (82073570), the Swedish Heart and Lung Foundation (20180306), the Karolinska Institutet’s Research Foundation (2018-01547), and the Swedish Council for Working Life and Social Research (2015-00837).

## Conflict of Interest

The authors declare that the research was conducted in the absence of any commercial or financial relationships that could be construed as a potential conflict of interest.

## Publisher’s Note

All claims expressed in this article are solely those of the authors and do not necessarily represent those of their affiliated organizations, or those of the publisher, the editors and the reviewers. Any product that may be evaluated in this article, or claim that may be made by its manufacturer, is not guaranteed or endorsed by the publisher.
