# Supplementary material for: Association of Maternal Hypothyroidism With Cardiovascular Diseases in the Offspring
Source: Front Endocrinol (Lausanne). 2021 Aug 31;12:739629. doi: 10.3389/fendo.2021.739629 (PMC8438132; doi:10.3389/fendo.2021.739629)
Supplement: Supplementary file 1 [file DataSheet_1.docx]

**Contents of Supplementary Files**

**Supplementary Table 1.** International Classification of Diseases codes for the main subcategories of cardiovascular diseases.

**Supplementary Table 2.** The distribution of the main diagnoses at first maternal hypothyroidism diagnosis.

**Supplementary Figure S1.** The incidence rates of CVD and hypertension among children exposed to maternal hypothyroidism and unexposed

**Supplementary Figure S2.** The association between maternal hypothyroidism and offspring’s CVD stratified by maternal diabetes.

**Supplementary Figure S3.** The association between maternal hypothyroidism and offspring’s CVD stratified by maternal education at childbirth.

**Supplementary Figure S4.** The association between maternal hypothyroidism and offspring’s CVD stratified by maternal age at delivery.

**Supplementary** **Figure S5.** The association between maternal hypothyroidism and offspring’s CVD stratified by gender of children.

**Supplementary Figure S6.** The association between maternal hypothyroidism and offspring’s CVD stratified by age of CVD diagnosis.

**Supplementary Table 1. International Classification of Diseases codes for the main subcategories of cardiovascular diseases**

|  | **ICD-10** | **ICD-8** |
| --- | --- | --- |
| Hypertension | I10-I15 | 400-404, |
| Ischemic heart disease | I20-I25 | 410-414 |
| Arrhythmia | I44-I49 | 4272-4279 |
| Supraventricular arrhythmias | I471, I478, I479, I48 | 42790, 42793, 42794 |
| Ventricular arrhythmias | I46, I470, I472, I490 | 42791, 52797 |
| Atrial fibrillation | I48 | 4274 |
| Stroke | I60-I69 | 430-438 |
| Acute myocardial infarction | I21-I22 | 410-411 |

**Supplementary Table 2. The distribution of the main diagnoses at first maternal hypothyroidism diagnosis**

| **Disease categories** | **ICD-8** | **ICD-10** | **During Pregnancy** | **After Delivery** |
| --- | --- | --- | --- | --- |
| Certain infectious and parasitic diseases | 000-139 | A00-B99 | 0.00% | 1.83 % |
| Neoplasms | 140-239 | C00-D48 | 0.00% | 7.22 % |
| Diseases of the blood and blood-forming organs | 280-289 | D50-D89 | 0.00% | 1.03 % |
| Endocrine, nutritional, and metabolic disease | 240-279 | E00-E90 | 23.18% | 23.16 % |
| Hypothyroidism | 243-244 | E03, E89.0 | 19.92% | 15.73% |
| Mental and behavioral disorders | 290-319 | F00-F99 | 0.20% | 1.06 % |
| Diseases of the nervous system | 320-359 | G00-G99 | 0.40% | 1.99 % |
| Diseases of the eye and adnexa | 360-379 | H00-H59 | 0.00% | 0.45 % |
| Diseases of the ear and mastoid process | 380-389 | H60-H95 | 0.00% | 0.25 % |
| Diseases of the circulatory system | 390-459 | I00-I99 | 0.20% | 6.01 % |
| Diseases of the respiratory system | 460-519 | J00-J99 | 1.00% | 4.70 % |
| Diseases of the digestive system | 520-579 | K00-K93 | 0.41% | 10.88 % |
| Diseases of the skin and subcutaneous tissue | 680-709 | L00-L99 | 0.20% | 1.13 % |
| Musculoskeletal and connective tissue diseases | 710-739 | M00-M99 | 0.00% | 4.32 % |
| Diseases of the genitourinary system | 580-629 | N00-N99 | 0.40% | 10.74 % |
| Pregnancy, childbirth related disorders | 630-679 | O00-O99 | 68.88% | 5.99 % |
| Single spontaneous delivery | 650 | O80 | 27.84% | 0% |
| Conditions originating in the perinatal period | 760-779 | P00-P96 | 0.00% | 0.00 % |
| Congenital malformations and deformations | 740-759 | Q00-Q99 | 0.20% | 0.14 % |
| Symptoms, signs, and findings not classified elsewhere | 780-799 | R00-R99 | 0.61% | 7.98 % |
| External causes of injury and poisoning | 800-999 | S00-T98 | 0.81% | 3.88% |
| External causes of morbidity and mortality | E00-E99 | X01-Y98 | 2.23% | 0.10 % |
| Factors influencing health and contact with health services | Y00-Y99 | Z00-Z99 | 1.21% | 8.45 % |

**
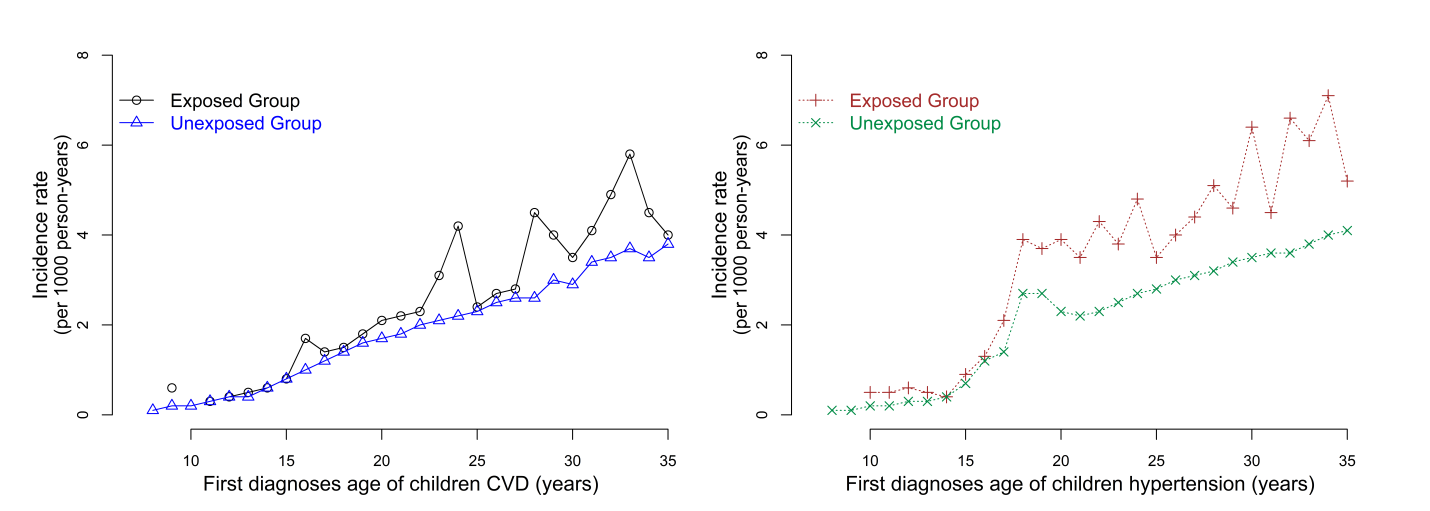
**

**Supplementary Figure S1. The incidence rates of CVD and hypertension (identified through patient registry and prescription registry) among children exposed to maternal hypothyroidism and unexposed**

**
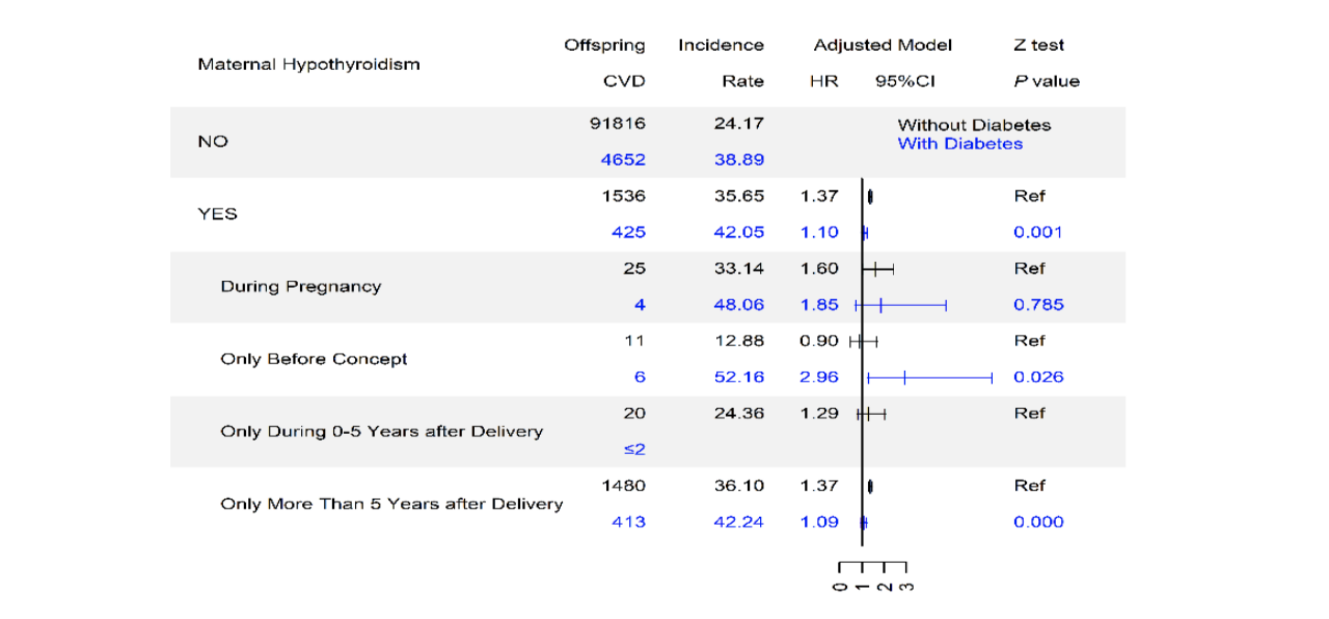
**

**Supplementary Figure S2. The association between maternal hypothyroidism and offspring’s CVD stratified by maternal diabetes.**

Abbreviation: CVD, cardiovascular disease; Incidence rate, incidence rate per 1000 person-years; HR, hazard ratio; CI, confidence interval; Ref, reference group

**

**

**Supplementary Figure S3. The association between maternal hypothyroidism and offspring’s CVD stratified by maternal education at childbirth.**

Abbreviation: CVD, cardiovascular disease; Incidence rate, incidence rate per 1000 person-years; HR, hazard ratio; CI, confidence interval; Ref, reference group

**

**

**Supplementary Figure S4. The association between maternal hypothyroidism and offspring’s CVD stratified by maternal age at delivery.**

Abbreviation: CVD, cardiovascular disease; Incidence rate, incidence rate per 1000 person-years; HR, hazard ratio; CI, confidence interval; Ref, reference group

**

**

**Supplementary Figure S5. The association between maternal hypothyroidism and offspring’s CVD stratified by gender of children.**

Abbreviation: CVD, cardiovascular disease; Incidence rate, incidence rate per 1000 person-years; HR, hazard ratio; CI, confidence interval; Ref, reference group

**

**

**Supplementary Figure S6. The association between maternal hypothyroidism and offspring’s CVD stratified by age of CVD diagnosis.**

Abbreviation: CVD, cardiovascular disease; Incidence rate, incidence rate per 1000 person-years; HR, hazard ratio; CI, confidence interval; Ref, reference group
